# Use, timing and factors associated with tracheal intubation in septic shock: a prospective multicentric observational study

**DOI:** 10.1186/s13613-020-00668-6

**Published:** 2020-05-24

**Authors:** C. Darreau, F. Martino, M. Saint-Martin, S. Jacquier, J. F. Hamel, M. A. Nay, N. Terzi, G. Ledoux, F. Roche-Campo, L. Camous, F. Pene, T. Balzer, F. Bagate, J. Lorber, P. Bouju, C. Marois, R. Robert, S. Gaudry, M. Commereuc, M. Debarre, N. Chudeau, P. Labroca, K. Merouani, P. Y. Egreteau, V. Peigne, C. Bornstain, E. Lebas, F. Benezit, S. Vally, S. Lasocki, A. Robert, A. Delbove, N. Lerolle

**Affiliations:** 1 Medical and Surgical Intensive Care Unit, Le Mans Hospital, Le Mans, France; 2 Medical and Surgical Intensive Care Unit, Guadeloupe University Hospital, Les Abymes, Guadeloupe France; 3 Medical Intensive Care Unit, Tours University Hospital, Tours, France; 4 Methodology and Statistics Department, Angers University Hospital, Angers, France; 5 Medical Intensive Care Unit, Orleans Regional Hospital, Orléans, France; 6 Medical Intensive Care Unit, Grenoble University Hospital, La Tronche, France; 7 Medical and Surgical Intensive Care Unit, Lille University Hospital, Lille, France; 8 Intensive Care Unit, Hospital Verge de la Cinta, Tortosa, Spain; 9 Medical Intensive Care Unit, Bicêtre Hospital, AP-HP, Paris, France; 10 Medical Intensive Care Unit, Cochin Hospital, AP-HP, Paris, France; 11 Medical Intensive Care Unit, Brest University Hospital, Brest, France; 12 Medical Intensive Care Unit, Henri Mondor Hospital, AP-HP, Paris, France; 13 Medical and Surgical Intensive Care Unit, La-Roche-sur-Yon Hospital, La Roche-sur-Yon, France; 14 Medical and Surgical Intensive Care Unit, Sud Bretagne Hospital, Lorient, France; 15 Medical Intensive Care Unit, Pitié-Salpétrière Hospital, AP-HP, Paris, France; 16 Medical Intensive Care Unit, Poitiers University Hospital, Poitiers, France; 17 Medical Intensive Care Unit, Louis Mourier Hospital, AP-HP, Colombes, France; 18 Medical Intensive Care Unit, Georges Pompidou European Hospital, AP-HP, Paris, France; 19 Medical and Surgical Intensive Care Unit, Saint Brieuc Hospital, Saint Brieuc, France; 20 Medical Intensive Care Unit, Nancy University Central Hospital, Nancy, France; 21 Medical and Surgical Intensive Care Unit, Alençon Hospital, Alençon, France; 22 Medical and Surgical Intensive Care Unit, Morlaix Hospital, Morlaix, France; 23 Medical and Surgical Intensive Care Unit, Métropole Savoie Hospital, Chambéry, France; 24 Medical Intensive Care Unit, Le Raincy-Montfermeil Hospital, Montfermeil, France; 25 Medical and Surgical Intensive Care Unit, Bretagne Atlantique Hospital, Vannes, France; 26 Medical and Surgical Intensive Care Unit, Rennes University Hospital, Rennes, France; 27 Medical and Surgical Intensive Care Unit, Martinique University Hospital, Fort-de-France, Martinique France; 28 Surgical Intensive Care Unit, Angers University Hospital, Angers, France; 29 Medical Intensive Care Unit, Nice University Hospital, Nice, France; 30 Medical Intensive Care Unit, Nantes University Hospital, Nantes, France; 31 Medical Intensive Care Unit, Angers University Hospital, Angers, France

**Keywords:** Septic shock, Tracheal intubation, Mechanical ventilation

## Abstract

**Background:**

No recommendation exists about the timing and setting for tracheal intubation and mechanical ventilation in septic shock.

**Patients and methods:**

This prospective multicenter observational study was conducted in 30 ICUs in France and Spain. All consecutive patients presenting with septic shock were eligible. The use of tracheal intubation was described across the participating ICUs. A multivariate analysis was performed to identify parameters associated with early intubation (before H8 following vasopressor onset).

**Results:**

Eight hundred and fifty-nine patients were enrolled. Two hundred and nine patients were intubated early (24%, range 4.5–47%), across the 18 centers with at least 20 patients included. The cumulative intubation rate during the ICU stay was 324/859 (38%, range 14–65%). In the multivariate analysis, seven parameters were significantly associated with early intubation and ranked as follows by decreasing weight: Glasgow score, center effect, use of accessory respiratory muscles, lactate level, vasopressor dose, pH and inability to clear tracheal secretions. Global *R*-square of the model was only 60% indicating that 40% of the variability of the intubation process was related to other parameters than those entered in this analysis.

**Conclusion:**

Neurological, respiratory and hemodynamic parameters only partially explained the use of tracheal intubation in septic shock patients. Center effect was important. Finally, a vast part of the variability of intubation remained unexplained by patient characteristics.

*Trial registration* Clinical trials NCT02780466, registered on May 23, 2016. https://clinicaltrials.gov/ct2/show/NCT02780466?term=intubatic&draw=2&rank=1.

## Background

Several international guidelines such as the Surviving Sepsis Campaign help physicians to manage septic shock patient [1]. However, these guidelines do not indicate the place for tracheal intubation and initiation of mechanical ventilation.

Several arguments have been put forward in favor of early ventilatory support in septic shock patients, as part of the bundle that should be introduced in the first hours of care together with antibiotic, fluid, and vasopressor use. Reducing work of breathing and oxygen consumption to maintain adequate tissue oxygenation, preventing diaphragmatic dysfunction and self-inflicted lung injury are expected benefits of invasive ventilation [2, 3]. Several studies have shown that diaphragmatic dysfunction may occur as early as in the first 4 h of septic shock [4, 5]. On the other hand, arguments to challenge a systematic use of ventilatory support in septic shock include immediate complications of tracheal intubation (including hemodynamic impairment) as well as potential side effects related to ventilation (ventilator-induced diaphragm dysfunction, muscle atrophy, ventilation-induced lung injuries, and ventilator-associated pneumonia) [6–8]. In recent multicenter trials on septic shock patients, the percentage of patients who received invasive ventilation ranged widely from 40 to 80% [9, 10]. In a declarative international survey, 86% of clinicians declared that the decision to initiate invasive ventilation was based on “common sense” or “human physiological data” [11]. Agreement between responders regarding hemodynamic parameters to initiate mechanical ventilation was low, contrary to neurologic and respiratory criteria. In acute neurologic and respiratory failures, indications for intubation and mechanical ventilation are generally well-accepted across studies, based on reliable quantitative parameters (respiratory rate, use of accessory respiratory muscles, PaO_2_/FiO_2_ ratio, and Glasgow coma score) [12–16]. How physicians apply these criteria in septic shock patients and whether other parameters, in particular hemodynamic, are involved is unknown.

To assess use, timing and factors associated with tracheal intubation in septic shock patients, we conducted a multicenter observational prospective study in 30 intensive care units (ICUs) in France and Spain. Our hypothesis was that in the absence of specific recommendations in the sepsis care bundle, use and timing of tracheal intubation might vary greatly between patients.

## Methods

The INTUBATIC study was a prospective, multicenter observational study conducted in 30 ICUs (29 in France and one in Spain) in both academic and non-academic hospitals. The primary endpoint was to assess rate of intubation, early and late, and factors associated with intubation practice in septic shock patients. The secondary endpoint was to assess mortality according to intubation.

We considered a time window of 8 h following vasopressor onset to define early intubation, from the results of our previous study [17]. Two approaches were used to assess the parameters associated with early intubation. First, criteria for standard indications for early tracheal intubation were defined a priori, based on accepted recommendations for invasive ventilation initiation in neurologic or respiratory failure. Frequency of early intubation and patient characteristics were described among those with such criteria [12–16]. Second, a multivariate analysis entering a wide array of parameters was performed to assess the link between these parameters and early intubation among all patients. An evaluation of the goodness-of-fit of the model was performed to determine the amount of early intubation variability explained by the different covariates.

The inclusion period ran from May 2016 to October 2017. ICUs were involved gradually and had 12-months to include patients. The protocol allowed for a maximum of 60 patients enrolled per center. Physicians participating in the study were aware of its main objective, but were instructed to perform patient’s care as they usually did, and no specific hypothesis was put forward regarding optimal management of these patients. Patients, or a proxy if the patient was deemed unable to consent, received oral and written information about the study, and oral consent was obtained before inclusion. When a proxy gave the initial consent, the patient’s consent was further obtained whenever possible. This study was approved by the Angers University Hospital Ethics committee (n° 2015/96).

### Patients

Patients aged 18 years and above were eligible if admitted in a participating ICU for septic shock, defined by a documented or clinical suspicion of infection and hypotension requiring vasopressor infusion despite adequate fluid loading. Patients could be included if a vasopressor was introduced in the 24 h preceding ICU admission, i.e., in another hospital or the emergency room. Patients were not eligible if vasopressor infusion was started after tracheal intubation and mechanical ventilation (non-invasive ventilation through facial mask did not prevent inclusion). Incapacitated adults, pregnant women, patients with a decision of withdrawal or withholding of care before ICU admission, patients without social health insurance and patients who refused to participate in the study were not included.

### Measurements

Age, sex, and main comorbidities were noted. SAPS II and SOFA score were calculated 24 h after ICU admission [18, 19]. Infection site, causal pathogen(s), and nosocomial or community-acquired subset of infection were registered. Hemodynamic, respiratory, and neurological parameters were recorded between the time of vasopressor onset (H0) and H8: oxygen administration device and PaO_2_/FiO_2_ ratio (see Additional file 1: Table S1 for FiO_2_ determination), respiratory rate, accessory inspiratory muscle use, paradoxical abdominal breathing, inability to cough or to clear tracheal secretions, vasopressor infusion rate (norepinephrine or epinephrine in all centers), arterial lactate concentration, serum creatinine level, urine output, and Glasgow coma score. In patients not intubated by H8, the worst values of acute neurological and respiratory severity parameters over this period were registered. In patients intubated before H8, the worst parameters between H0 and intubation were registered. Fluid loading from the first hypotension to H0 was noted. Durations of ICU stay, hospital stay, vasopressor infusion, mechanical ventilation, and renal replacement therapy were registered. ICU, hospital and 28-day mortality were recorded.

### Standard criteria for theoretical immediate intubation

Criteria qualifying for theoretical immediate tracheal intubation during the H0–H8 time window were either (independently of intubation being performed or not):

- Neurological failure: Glasgow coma scale < 10.
- Respiratory failure, two criteria among these had to be present: oxygen saturation less than 90% during more than 5 min despite optimized oxygen administration, respiratory rate more than 35 per minute, significant accessory respiratory muscle use, respiratory acidosis defined by pH < 7.35 and pCO_2_ > 45 mmHg, hypoxemia with PaO_2_/FiO_2_ ratio inferior to 150, and inability to cough or clear tracheal secretions. These criteria are generally associated with strong recommendations for immediate tracheal intubation in case of neurological or respiratory failure [12–16].

### Statistical analyses

We sought to include more than 800 patients, estimating an early intubation rate ranging from 20 to 40%. This allowed for the selection of 20 to 25 covariates to be included in a multivariable model for explaining early intubation [20]. Continuous data were summarized as the mean and standard deviation or median with inter-quartile range as required and compared using the Kruskal–Wallis test. Categorical data were expressed as number and percentage and compared using the Fisher exact test. The cumulative hazard function of event occurrence was estimated using the Nelson–Aalen procedure.

Early intubation was studied through a mixed-effects logistic regression model, considering a list of covariates (considered as fixed effect covariates). Covariates entered in the multivariate analysis were chosen a priori based on their relevance after reaching a consensus between NL, JFH, AD, MSM, FM, SJ and CD based on published literature review and advice from experts of the topics (Prof. Pierre Asfar and Prof. Laurent Brochard). The practice variation between participating centers was also considered in this model by including “center” as a random effect covariate. Patients intubated for emergent surgery procedure were excluded for this analysis. No imputation was performed for missing data. The goodness-of-fit was assessed using a McKelvey pseudo *R*-squared measure, evaluating the outcome variability based on the constructed model [21]. The weight of each covariate included in the outcome variability explanation was assessed through the percentage of McKelvey pseudo *R*-squared associated with this covariate [22].

Graphical representation of patients survival was performed with Kaplan–Meier method, and survival rates were compared with log-rank test. All the tests were two-sided considering a type I error set at 0.05. The statistical analyses were performed using Stata^®^ 13.1.

## Results

**Fig. 1:**
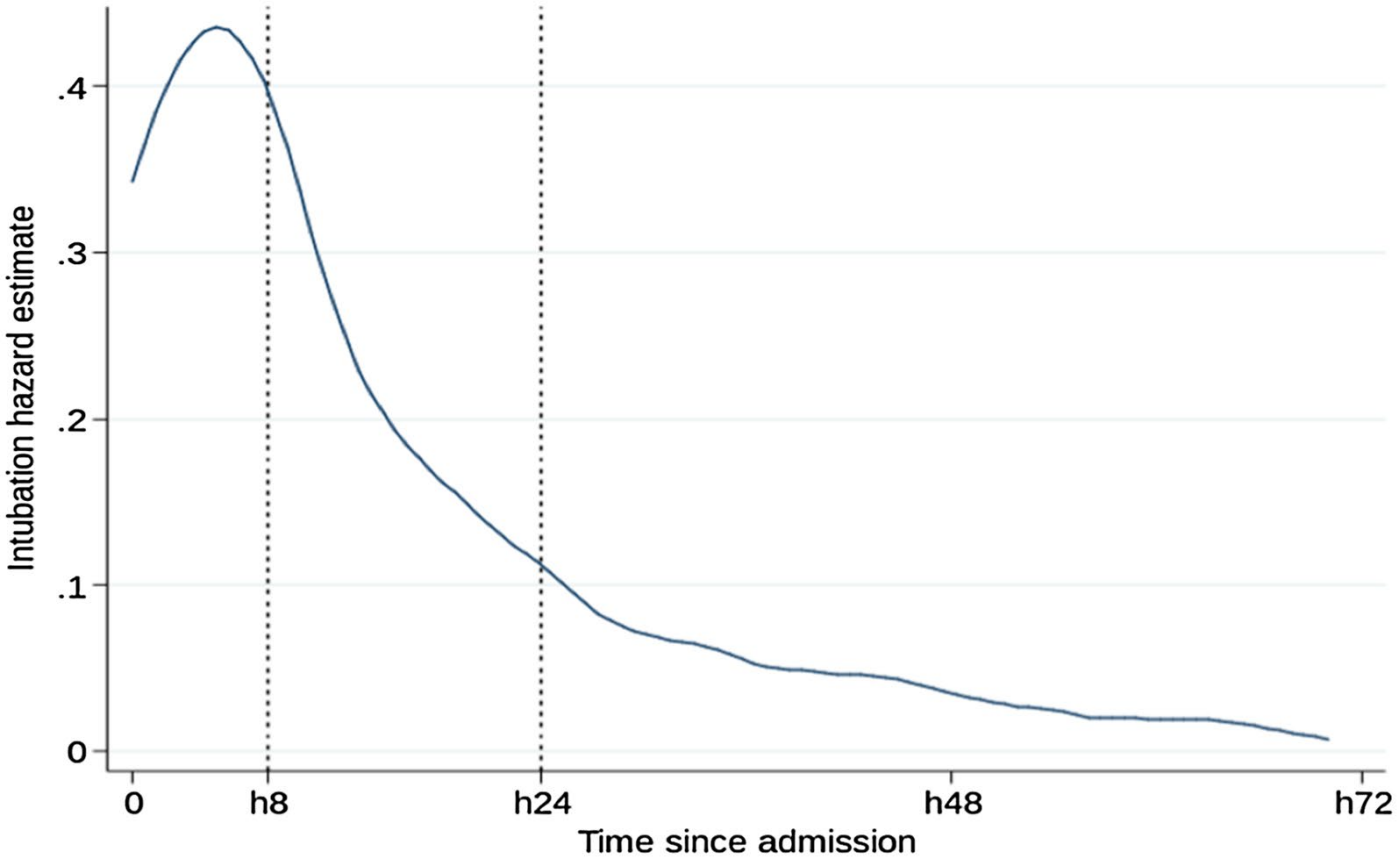
Hazard of being intubated from H0 to H72

Eight hundred and fifty-nine patients were enrolled in the study. Early intubation (i.e., in the 8 h following vasopressor initiation) was performed in 209 patients (24%). At 24 h, 70 additional patients had been intubated and 44 were intubated between H24 and H72, and only one thereafter during the ICU stay. Cumulative intubation rate over the ICU stay was therefore 324/859 (38%, range among the centers with at least 20 patients included 14–65%; Q1–Q3 27–46%). The hazard of being intubated over the first 72 h is displayed in Fig. 1.

**Fig. 2:**
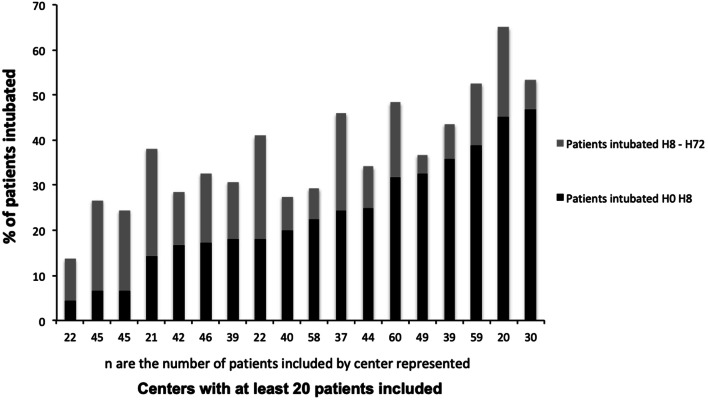
Percentage of patients intubated at H8 and H72 in the 18 centers with at last 20 patients included

The percentage of patients intubated early ranged from 4.5 to 47%, [Q1–Q3: 17.4–32.6%] across the 18 centers with at least 20 patients included (these centers included 718 patients), see Fig. 2.

Data of patients intubated early, late (after H8) and never intubated are displayed in Table 1: parameters associated with acute severity (i.e., respiratory and hemodynamic variables), chemotherapy, presence of fungus and some site of infections (digestive and urinary) were significantly different between patients intubated early, late, and never intubated.

**Table 1:** Patient’s demographic data and outcome according intubation during the ICU stay

	Patients intubated early H0–H8 (*n* = 209)	Patients intubated late H8–H72 (n = 114)	Patients never intubated (*n* = 536)	*P* value
Age, years	66.5 ± 13.9	64.6 ± 13.5	64.9 ± 14.6	0.30
Sex, female	82 (39.4%)	35 (30.7%)	196 (36.6%)	0.30
Weight, kg	77.1 ± 18.7	76.7 ± 18.8	75.9 ± 19.9	0.32
Chronic heart failure, NYHA > 2	15 (7.2%)	11 (9.7%)	55 (10.3%)	0.43
Chronic respiratory failure	33 (15.8%)	16 (14.2%)	84 (15.7%)	0.93
Home oxygen therapy	4 (1.9%)	3 (2.6%)	6 (1.1%)	0.33
Home non-invasive or invasive ventilation therapy	2 (1%)	4 (3.5%)	7 (1.3%)	0.18
Chronic renal failure	32 (15.3%)	15 (13.2%)	88 (16.5%)	0.72
Dialysis	10 (4.8%)	2 (1.8%)	29 (5.4%)	0.28
Cirrhosis	24 (11.5%)	6 (5.3%)	36 (6.7%)	0.07
Immunosuppression	51 (24.4%)	40 (35.4%)	161 (30.3%)	0.12
Chemotherapy	26 (12.4%)	30 (36.3%)	80 (14.9%)	0.02
Corticosteroid therapy > 20 mg/j	12 (5.7%)	10 (8.8%)	41 (7.6%)	0.98
Organ transplant or bone marrow transplant	11 (5.3%)	5 (4.4%)	36 (6.7%)	0.37
Human immunodeficiency virus	6 (2.7%)	0 (0.00%)	13 (2.4%)	0.07
Other immunosuppressive therapy	11 (5.3%)	8 (7%)	46 (8.6%)	0.44
Neutropenia < 500/mm^3^	18 (13.4%)	13 (11.4%)	37 (6.8%)	0.13
Infection site				
Heart	5 (2.4%)	5 (4.6%)	10 (2%)	0.27
Skin	26 (12.4%)	12 (11%)	55 (10.8%)	0.69
Digestive	62 (29.7%)	48 (43.2%)	136 (26.8%)	0.01
Gynecological	3 (1.4%)	0 (0.00%)	1 (0.2%)	0.09
Material	10 (7.8%)	7 (6.5%)	32 (6.4%)	0.83
Neurological	0 (0.00%)	1 (0.9%)	1 (0.2%)	0.31
Upper respiratory tract	4 (1.9%)	2 (1.9%)	7 (1.4%)	0.65
Bone	2 (1%)	1 (0.9%)	5 (1%)	0.99
Lung	59 (28.2%)	29 (26.9%)	124 (24.2%)	0.37
Blood	55 (27.6%)	35 (31.8%)	129 (25.5%)	0.37
Urinary	37 (18.6%)	9 (8.3%)	144 (28.1%)	< 0.001
Others	8 (4%)	2 (1.9%)	14 (2.8%)	0.73
Pathogens				
Gram-positive cocci	61 (31.1%)	35 (32.4%)	122 (24%)	0.06
Gram-negative bacilli	104 (49.8%)	53 (48.6%)	243 (47.3%)	0.52
Fungus, parasite	9 (4.6%)	3 (2.8%)	6 (1.2%)	0.02
Others	9 (4.6%)	5 (4.7%)	20 (4%)	0.84
Non-identified	46 (23.6%)	29 (26.9%)	162 (31.6%)	0.1
Infection type				
Nosocomial infection	147 (71%)	64 (56.6%)	329 (61.6%)	0.08
Community-acquired infection	20 (9.7%)	17 (15%)	75 (14%)	
Healthcare associated infection	40 (19.3%)	32 (28.3%)	130 (24.3%)	
Acute physiological parameters and treatment				
pH^a^	7.26 ± 0.15	7.33 ± 0.1	7.38 ± 0.1	< 0.001
PaO_2_, mmHg^a^	103.8 ± 54.2	87.9 ± 29.0	93.8 ± 30.1	0.16
PaCO_2_, mmHg^a^	36.5 ± 14.5	32.2 ± 8.8	32.3 ± 8.8	0.02
SpO_2_, %^a^	93.6 ± 5.9	92.3 ± 6.4	95.2 ± 3.3	0.03
Lactates^a^	5.4 ± 4.0	3.5 ± 2.3	2.9 ± 2.3	< 0.001
Respiratory rate^a^	28.9 ± 7.9	28.6 ± 6.6	25.7 ± 6.7	< 0.001
Inability to clear tracheal secretions^a^	48 (23.7%)	13 (11.6%)	40 (7.6%)	< 0.001
Use of accessory respiratory muscle^a^	101 (49.8%)	22 (19.8%)	52 (9.9%)	< 0.001
Standard nasal oxygen^b^	70 (35.7%)	53 (49.1%)	273 (52.8%)	< 0.001
High concentration mask oxygen^b^	76 (39.2%)	17 (15.6%)	55 (10.9%)	< 0.001
High-flow nasal therapy^b^	22 (11.4%)	21 (19.6%)	41 (8.1%)	< 0.01
Non-invasive ventilation therapy^b^	20 (10.4%)	8 (7.6%)	26 (5.2%)	0.04
PaO_2_/FiO_2_ ratio^a^	182 (88–295)	192 (127–313)	295 (208–395)	< 0.001
Glasgow coma score^a^	12.4 ± 3.9	14.5 ± 1.5	14.7 ± 0.9	< 0.001
Vasopressor dose^a^, µg/kg/min	0.74 ± 0.66	0.50 ± 0.46	0.35 ± 0.37	< 0.001
Cumulative fluid from first hypotension to H0	2218.8 ± 1243.2	1958.6 ± 1137.5	2179.8 ± 1327.1	0.25
SAPS II score at 24 h	67.9 ± 22.0	58.8 ± 18.8	46.9 ± 13.8	< 0.001

Data are n (%) or mean ± SD

^a^Worst value recorded between H0 and H8, or between H0 and immediately before intubation if intubation performed before H8

^b^At H0

**Fig. 3:**
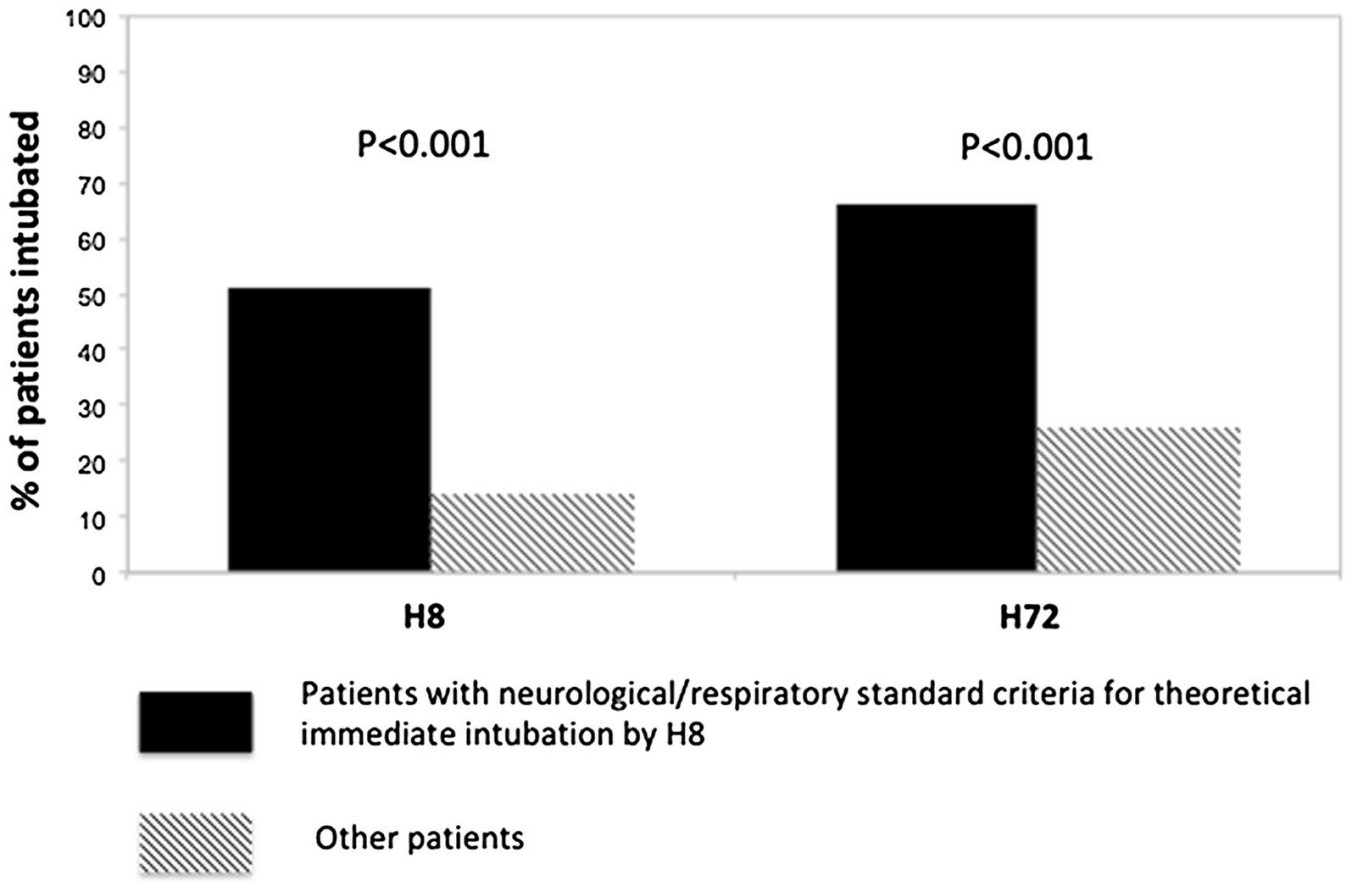
Intubation frequency at H8 and H72 in patients with and without neurological or respiratory standard criteria for theoretical immediate intubation by H8

Two hundred and twenty-six patients reached neurological or respiratory standard criteria for theoretical immediate intubation by H8 and 519 patients did not reach such criteria by H8. Figure 3 shows frequency of intubation at H8 and H72 in these two subgroups of patients, showing that among patients with neurological or respiratory standard criteria for theoretical immediate intubation by H8, only 51% were intubated at this time (66% by H72). One hundred and fourteen patients could not be classified due to missing data. In the group of patients with standard criteria for theoretical immediate intubation, patients intubated early had more severe respiratory, hemodynamic and neurologic parameters in comparison with patients not intubated before H8 and had a worse outcome when considering the number of days without organ support and survival (see Additional file 1: Table S2).

Predictor variables used in the mixed-effects logistic regression model are detailed in Table 2 and its footnote. According to physicians, 71 (8.3%) patients were intubated for emergent surgical procedures; these patients were excluded from the dataset for the analysis. Seven parameters were significantly associated with intubation by H8 and ranked in the model as follows by decreasing weight: Glasgow score, center effect, use of accessory respiratory muscles, lactate, vasopressor dose, pH and inability to clear tracheal secretions. However, the global *R*-square of the model was only 60% indicating that 40% of the variance of the decision to intubate or not was related to other variables than those entered in this analysis.

**Table 2:** Multivariate analysis for explaining early intubation

	Odds ratio [95% confidence interval]	*P*-value	% of *r*^2^ contribution	Covariate rank in *r*^2^ contribution
Use of accessory respiratory muscles^a^	5.63 [2.83–11.82]	< 0.001	12.8	3
pH (for each 0.1 decrease)^a^	1.51 [1.09–2.97]	0.02	3.8	7
Glasgow score (reference ≥ 14)^a^			39.5	1
10–13 vs ≥ 14	3.03 [1.20–7.63]	0.01		
<10 vs ≥ 14	39.95 [10.13–134.85]	< 0.001		
Inability to clear tracheal secretions^a^	2.64 [1.38–3.96]	0.02	6.02	6
Vasopressor (for each 1 μg/kg/min increase)^a^	2.34 [1.38–3.96]	0.001	5.7	5
Lactate (for each 1 mmol/L increase)^a^	1.11 [1.01–1.21]	0.02	8.6	4

	Variance estimate [95% confidence interval]			
Variable effect: center	0.50 [0.12–1.81]	< 0.01	16.3	2

Overall *r*^2^ of the model was 0.6

Parameters entered in the model not presented in the table, with a *P*-value > 0.1: age, sex, weight, NYHA status, chronic respiratory failure, chronic renal failure, immunosuppression, pulmonary and urinary site of infection, pathogen, respiratory rate, PaCO_2_, PaO_2_/FiO_2_ ratio, fluid loading from first hypotension to H0

^a^Worst value recorded between H0 and H8, or between H0 and immediately before intubation if performed before H8

**Fig. 4:**
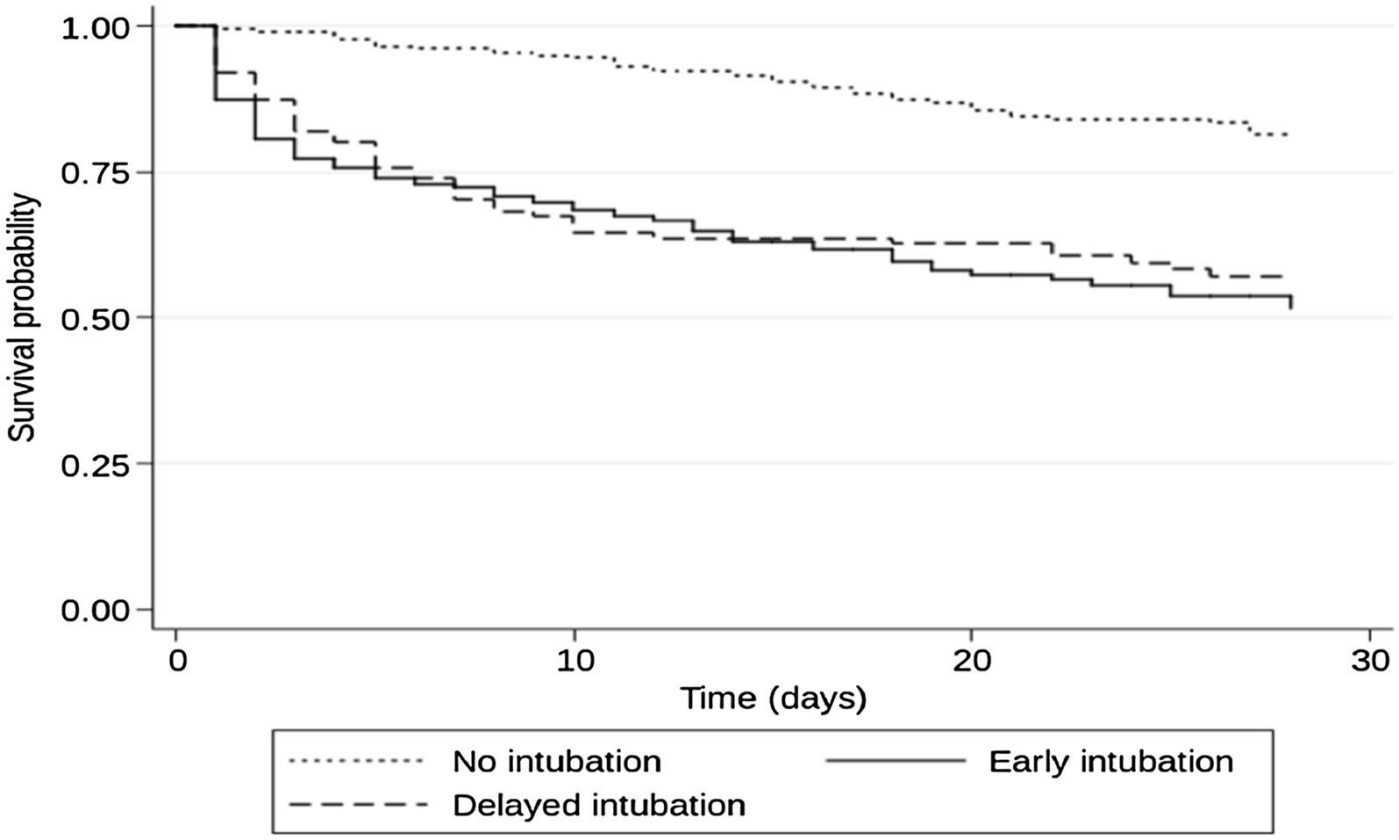
28-day survival according to intubation status. *P* = 0.87 for comparison between patients intubated early vs. delayed, and *P* = 0.001 for comparisons between patients intubated early vs. never intubated

Finally, mortality and other outcomes were significantly different between patients intubated early, late, and never intubated (see Table 3). Survival according to intubation status is displayed in Fig. 4. Patients never intubated had the higher survival and better outcomes. Comparison of patients intubated early vs. late showed no survival difference, but longer length of ICU and hospital stay in these latter patients.

**Table 3:** Patient’s outcome according to intubation during the ICU stay

	Patients intubated early H0–H8 (*n* = 209)	Patients intubated late H8–H72 (*n* = 114)	Patients never intubated (*n* = 536)	Overall *P*-value	Intubated early vs. late *P*-value
Number of days alive without vasopressor at day 28	13.7 ± 14.3	13.7 ± 11.4	24.3 ± 17.5	< 0.001	0.99
Number of days alive without dialysis at day 28	15.8 ± 14.7	17.4 ± 11.5	26.2 ± 17.4	<0.001	0.09
Number of days alive without invasive ventilation at day 28	11.5 ± 14.1	11.9 ± 10.5	26.3 ± 17.5	< 0.001	0.145
Length of ICU stay, days	14.7 ± 35.2	15.2 ± 20.4	5.1 ± 37.1	< 0.001	0.01
Length of hospital stay, days	22.7 ± 34.0	29.3 ± 41.0	21.7 ± 50.9	0.01	0.01
ICU mortality	97 (46.9%)	44 (38.6%)	32 (6%)	< 0.001	0.2
Hospital mortality	106 (51%)	56 (49.1%)	75 (14.1%)	< 0.001	0.8
28th-day mortality	97 (46.9%)	47 (41.2%)	70 (13.1%)	< 0.001	0.4

## Discussion

In this observational study conducted over 859 patients in 30 ICUs, early intubation (in the 8 h following vasopressor onset) was performed in 24% of the patients. Thirty-eight percent were intubated at H72. A wide variation was observed across centers. In the patients with standard criteria usually recognized for immediate tracheal intubation due to neurological and/or respiratory failure before H8, only half of them were intubated early [12–16]. In a multivariate analysis entering a wide array of parameters, seven neurological, respiratory and hemodynamics acute severity parameters were associated with early intubation. Center effect was also prominent. Finally, the model only explained 60% of the variance of early intubation, meaning that 40% was related to unmeasured parameters. The survival curves showed no mortality difference between early and delayed intubation. Conversely, never-intubated patients had better survival.

Few studies have specifically evaluated tracheal intubation in septic shock patients. From large interventional studies on septic shock, although not designed to study intubation specifically, a trend towards lesser intubation rates can be observed, from 80% in the 2000–2011 period to 30% in recent years, which is in line with the rates observed in our study [10, 23, 24]. In a monocentric qualitative survey, Bauer et al. analyzed the factors that influenced the decision of early intubation in sepsis-associated respiratory failure. They showed that the decision to intubate was not solely based on clinical parameters, but also on clinician factors like background, experience or instinct and system factors like organizational structure or workload [25]. Our study confirms and extends these data by showing quantitatively that a vast component of the decision process to intubate in septic shock patients is not related to acute severity parameters or baseline conditions.

Initiating tracheal intubation and mechanical ventilation in a septic shock patient is not an inconsequential decision with potential harms and benefit as pointed out in the introduction. To this date, it is impossible to determine whether the place given to tracheal intubation in septic shock patients is adequate or not. The model used in this study only displays the parameters probably taken into account in the decision process, but not if these parameters are appropriate or not for the patient’s outcome. Although we did not observe any difference in mortality between patients intubated early and late, we cannot conclude on the adequate timing for this procedure. Indeed, the fact that our model only explains half of the variance leaves a wide range of parameters beyond the scope of our analysis. Those unknown and possibly important parameters make the impact of intubation and its timing on outcome difficult to analyze in a multivariable model or a propensity score model.

Our study has several limitations. The 8-h threshold for defining early intubation could be discussed. As already cited, a previous study showed that tracheal intubation was mostly performed during the first 6 h of septic shock [17]. Six hours is an accepted time frame for early intervention in sepsis [26]. To take into account the workload requirements and organizational factors of this demanding procedure, we extended to the first 8 h the time frame for defining early tracheal intubation. Missing data were observed in 13% of included patients, which may impact the accuracy of our results. We may have missed some important parameters that should have been considered to explain the decision to intubate. However, the prospective and dedicated design of our study allowed the monitoring of a wide range of baseline and clinical parameters. We may also have missed the adequate time frame during which parameters should have been monitored. In a future study, organizational factors (physician experience, workload, etc.) may be registered. Another hypothesis is that this uncertainty represents clinical equipoise, opening the way for a randomized trial comparing early (8 h) vs. late (rescue) tracheal intubation.

## Conclusion

In conclusion, we observed that 24% of patients with septic shock were intubated by H8 and 38% at H72. In a multivariate analysis, neurological, respiratory and hemodynamic parameters were associated with early intubation. Center effect had a strong influence on the model and, an important part of the variance remained unexplained. A significant part of the decision to intubate seemed independent of patient characteristics.

## Supplementary information

**Additional file 1: Table S1.** Conversion table for FiO_2_ determination. **Table S2.** Comparison between patients intubated early and not intubated early in the group of patients with standard criteria for early endotracheal intubation.

## Data Availability

The datasets used and analyzed during the current study are available from the corresponding author on reasonable request.

## Abbreviations

ICU: Intensive care unit
PaO2: Arterial oxygen partial pressure
FIO2: Inspired oxygen fraction
SAPS II: Simplified Acute Physiologic parameter
SOFA: Sequential Organ Failure Assessment
VO2: Oxygen consumption
NYHA: New York Heart Association functional classification
PaCO_2_: Arterial carbon dioxide partial pressure
SpO_2_: Oxygen pulsed saturation

## References

[1] Dellinger RP, Carlet JM, Masur H, Gerlach H, Calandra T, Cohen J (2004). Surviving Sepsis Campaign guidelines for management of severe sepsis and septic shock. Crit Care Med.

[2] Ebihara S, Hussain SNA, Danialou G, Cho W-K, Gottfried SB, Petrof BJ (2002). Mechanical ventilation protects against diaphragm injury in sepsis: interaction of oxidative and mechanical stresses. Am J Respir Crit Care Med.

[3] Hussain SN, Roussos C (1985). Distribution of respiratory muscle and organ blood flow during endotoxic shock in dogs. J Appl Physiol.

[4] Brochard L, Slutsky A, Pesenti A (2017). Mechanical ventilation to minimize progression of lung injury in acute respiratory failure. Am J Respir Crit Care Med.

[5] Lanone S, Taillé C, Boczkowski J, Aubier M (2005). Diaphragmatic fatigue during sepsis and septic shock. Intensive Care Med.

[6] Hussain SN, Simkus G, Roussos C (1985). Respiratory muscle fatigue: a cause of ventilatory failure in septic shock. J Appl Physiol.

[7] Perbet S, De Jong A, Delmas J, Futier E, Pereira B, Jaber S (2015). Incidence of and risk factors for severe cardiovascular collapse after endotracheal intubation in the ICU: a multicenter observational study. Crit Care.

[8] Jaber S, Petrof BJ, Jung B, Chanques G, Berthet J-P, Rabuel C (2011). Rapidly progressive diaphragmatic weakness and injury during mechanical ventilation in humans. Am J Respir Crit Care Med.

[9] Quenot J-P, Binquet C, Kara F, Martinet O, Ganster F, Navellou J-C (2013). The epidemiology of septic shock in French intensive care units: the prospective multicenter cohort EPISS study. Crit Care.

[10] The PRISM Investigators (2017). Early, goal-directed therapy for septic shock—a patient-level meta-analysis. N Engl J Med.

[11] de Montmollin E, Aboab J, Ferrer R, Azoulay E, Annane D (2016). Criteria for initiation of invasive ventilation in septic shock: an international survey. J Crit Care.

[12] Antonelli M, Conti G, Moro M, Esquinas A, Gonzalez-Diaz G, Confalonieri M (2001). Predictors of failure of noninvasive positive pressure ventilation in patients with acute hypoxemic respiratory failure: a multi-center study. Intensive Care Med.

[13] Carrillo A, Gonzalez-Diaz G, Ferrer M, Martinez-Quintana ME, Lopez-Martinez A, Llamas N (2012). Non-invasive ventilation in community-acquired pneumonia and severe acute respiratory failure. Intensive Care Med.

[14] Ferrer M, Esquinas A, Leon M, Gonzalez G, Alarcon A, Torres A (2003). Noninvasive ventilation in severe hypoxemic respiratory failure: a randomized clinical trial. Am J Respir Crit Care Med.

[15] Frat J-P, Ragot S, Coudroy R, Constantin J-M, Girault C, Prat G (2018). Predictors of intubation in patients with acute hypoxemic respiratory failure treated with a noninvasive oxygenation strategy. Crit Care Med.

[16] Frat J-P, Thille AW, Mercat A, Girault C, Ragot S, Perbet S (2015). High-flow oxygen through nasal cannula in acute hypoxemic respiratory failure. N Engl J Med.

[17] Delbove A, Darreau C, Hamel JF, Asfar P, Lerolle N (2015). Impact of endotracheal intubation on septic shock outcome: a post hoc analysis of the SEPSISPAM trial. J Crit Care.

[18] Le Gall JR, Lemeshow S, Saulnier F (1993). A new Simplified Acute Physiology Score (SAPS II) based on a European/North American multicenter study. J Am Med Assoc.

[19] Vincent JL, Moreno R, Takala J, Willatts S, De Mendonça A, Bruining H (1996). The SOFA (Sepsis-related Organ Failure Assessment) score to describe organ dysfunction/failure. On behalf of the Working Group on Sepsis-Related Problems of the European Society of Intensive Care Medicine. Intensive Care Med.

[20] Peduzzi P, Concato J, Kemper E, Holford TR, Feinstein AR (1996). A simulation study of the number of events per variable in logistic regression analysis. J Clin Epidemiol.

[21] McKelvey RD, Zavoina W (1975). A statistical model for the analysis of ordinal level dependent variables. J Math Sociol.

[22] Hamel J-F, Sébille V, Challet-Bouju G, Hardouin J-B (2016). Partial credit model: estimations and tests of fit with Pcmodel. Stata J Promot Commun Stat Stata.

[23] Asfar P, Meziani F, Hamel J-F, Grelon F, Megarbane B, Anguel N (2014). High versus low blood-pressure target in patients with septic shock. N Engl J Med.

[24] Annane D, Aegerter P, Jars-Guincestre MC, Guidet B (2003). Current epidemiology of septic shock: the CUB-Réa Network. Am J Respir Crit Care Med.

[25] Bauer PR, Kumbamu A, Wilson ME, Pannu JK, Egginton JS, Kashyap R (2017). Timing of intubation in acute respiratory failure associated with sepsis: a mixed methods study. Mayo Clin Proc.

[26] Rivers E, Nguyen B, Havstad S, Ressler J, Muzzin A, Knoblich B (2001). Early goal-directed therapy in the treatment of severe sepsis and septic shock. N Engl J Med.

