# Supplementary material for: Use, timing and factors associated with tracheal intubation in septic shock: a prospective multicentric observational study
Source: Ann Intensive Care. 2020 May 24;10:62. doi: 10.1186/s13613-020-00668-6 (PMC7245631; doi:10.1186/s13613-020-00668-6)
Supplement: Supplementary file 1 — Additional file 1: Table S1. Conversion table for FiO2 determination. Table S2. Comparison between patients intubated early and not intubated early in the group of patients with standard criteria for early endotracheal intubation. [file 13613_2020_668_MOESM1_ESM.docx]

**Additional file 1**

**Table S1: Conversion table for Fi0_2_ determination**

| Administration device | O2 flow rate (L/min) | Estimated FiO_2_ (%) |
| --- | --- | --- |
| Nasal 02 | 1 | 24 |
|  | 2 | 28 |
|  | 3 | 32 |
|  | 4 | 36 |
|  | 5 | 40 |
|  | 6 | 44 |
| Facial Mask | 5 | 40 |
|  | 6-7 | 50 |
| High Concentration Mask | 6 | 60 |
|  | 7 | 70 |
|  | 8 | 80 |
|  | 9 | 90 |
|  | 10 | 95 |

**Table S2: Comparison between patients intubated early and not intubated early in the group of patients with standard criteria for early endotracheal intubation**

|  | Not intubated early (111) | Intubated early (115) | p-value |
| --- | --- | --- | --- |
| Age, years | 67.00 (58.00-75.00) | 65.50 (57.00-73.00) | 0,45 |
| Sex, female | 78 (70.27%) | 73 (64.04%) | 0,32 |
| Weight, Kg | 78.00 (64.00-88.00) | 76.00 (67.50-89.50) | 0,76 |
| Chronic heart failure, NYHA>2 | 15 (13.51%) | 8 (6.96%) | 0,13 |
| Chronic respiratory failure | 23 (20.91%) | 18 (15.65%) | 0,39 |
| Home oxygen therapy | 2 (1.80%) | 2 (1.74%) | 1,00 |
| Home non invasive ventilation | 3 (2.70%) | 0 (0.00%) | 0,12 |
| Chronic renal failure | 16 (14.41%) | 12 (10.43%) | 0,42 |
| Dialysis | 2 (1.80%) | 5 (4.35%) | 0,45 |
| Cirrhosis | 11 (10.00%) | 16 (13.91%) | 0,42 |
| Immunosuppression | 42 (37.84%) | 32 (28.32%) | 0,16 |
| Chemotherapy | 27 (64.29%) | 17 (53.13%) | 0,35 |
| Corticostroid therapy >20mg/j | 8 (19.51%) | 8 (26.67%) | 0,57 |
| Organ transplant or bone marrow transplant | 9 (21.95%) | 6 (20.00%) | 1,00 |
| Human immunodeficiency virus | 1 (2.44%) | 4 (13.33%) | 0,15 |
| Other immunosuppressive therapy | 13 (31.71%) | 7 (23.33%) | 0,59 |
| Neutropenia <500/mm3 | 16 (38.10%) | 10 (33.33%) | 0,80 |
| Infection Site |  |  |  |
| Heart | 2 (1.92%) | 4 (3.74%) | 0,68 |
| Skin and soft tissue | 10 (9.62%) | 10 (9.35%) | 1,00 |
| Digestive | 21 (19.81%) | 33 (30.56%) | 0,08 |
| Gynaecological | 0 (0.00%) | 2 (1.89%) | 0,50 |
| Material | 3 (2.88%) | 5 (4.72%) | 0,72 |
| Neurological | 0 (0.00%) | 0 (0.00%) | 0,00 |
| Upper respiratory tract | 0 (0.00%) | 1 (0.94%) | 1,00 |
| Bone | 1 (0.96%) | 1 (0.94%) | 1,00 |
| Lung | 61 (58.65%) | 48 (43.24%) | 0,03 |
| Blood | 25 (23.58%) | 23 (21.70%) | 0,87 |
| Urinary | 10 (9.43%) | 10 (9.35%) | 1,00 |
| Others | 1 (0.95%) | 5 (4.67%) | 0,21 |
| Pathogens |  |  |  |
| Gram-positive cocci | 27 (25.23%) | 42 (39.25%) | 0,04 |
| Gram-negative bacilli | 40 (37.04%) | 48 (44.04%) | 0,33 |
| Fungus, parasite | 0 (0.00%) | 5 (4.81%) | 0,03 |
| Others | 7 (6.67%) | 6 (5.77%) | 1,00 |
| Non-identified | 41 (38.32%) | 28 (26.67%) | 0,08 |
| Acute physiological parameters and treatments |  |  |  |
| pH* | 7.35 (7.27-7.41) | 7.25 (7.16-7.31) | <0,001 |
| PaO2*, mmHg | 79.00 (64.50-96.00) | 80.00 (65.00-114.00) | 0,42 |
| PaCO2*, mmHg | 34.00 (29.00-45.00) | 35.00 (27.00-48.00) | 1 |
| SpO2*, % | 93.00 (92.00-97.00) | 92.00 (89.00-96.00) | 0,15 |
| Lactate* | 2.60 (1.60-4.10) | 4.35 (2.80-7.40) | <0,001 |
| Respiratory rate* | 33.00 (27.00-38.00) | 30.00 (25.00-37.00) | 0,20 |
| Inability to clear tracheal secretions* | 31 (28.70%) | 45 (39.82%) | 0,09 |
| Use of accessory respiratory muscle* | 54 (50.00%) | 90 (79.65%) | <0,001 |
| Standard Nasal Oxygen £ | 33 (31.13%) | 25 (23.15%) | 0,22 |
| High concentrartion mask oxygen £ | 33 (30.56%) | 52 (49.06%) | 0,01 |
| High flow nasal therapy £ | 30 (28.30%) | 17 (16.04%) | 0,05 |
| Non invasive ventilation £ | 17 (16.04%) | 17 (16.19%) | 1 |
| PaO2/FiO2 ration * | 143 (100-222) | 106 (073-200) | 0,02 |
| Glasgow coma score* | 15.00 (14.00-15.00) | 13.50 (6.00-15.00) | <0,001 |
| Vasopressor dose*, µg/kg/min | 0.34 (0.20-0.63) | 0.59 (0.34-1.02) | <0,001 |
| SAPS II score at 24h | 50.00 (38.00-63.00) | 70.00 (53.00-89.00) | <0,001 |
| Outcome |  |  |  |
| 28th day mortality | 31 (28.70%) | 64 (56.14%) | <0,001 |

Data are n (%) or med (IQR)

* Worst value recorded between H0 and H8, or between H0 and immediately before intubation if intubation performed before H8.

£ At H0

**e-table 3: Multivariate analysis showing all parameters included in the model.**

|  | **Odds Ratio**  **[95% confidence interval]** | **P-value** | **%of r² contribution** | **Covariate rank in r^2^ contribution** |
| --- | --- | --- | --- | --- |
| Age (for each year-old increase) | 1.00 [0.99-1.02] | 0.56 |  |  |
| Sexe (female vs. male) | 1.35 [0.80-2.31] | 0.26 |  |  |
| Weight (for each kg increase) | 0.99 [0.99-1.01] | 0.68 |  |  |
| NYHA status >2 | 0.44 [0.16-1.20] | 0.11 |  |  |
| Chronic respiratory failure | 1.03 [0.50-2.11] | 0.94 |  |  |
| Chronic renal failure | 1.42 [0.68-2.98] | 0.35 |  |  |
| Cirrhosis | 1.34 [0.55-3.27] | 0.53 |  |  |
| Any form of immunosuppression | 0.94 [0.51-1.73] | 0.84 |  |  |
| Pulmonary site of infection | 0.52 [0.26-1.1] | 0.06 |  |  |
| Urinary site of infection | 1.06 [0.54-2.09] | 0.85 |  |  |
| Pathogen is gram-positive cocci (vs. gram negative bacilli) | 0.74 [0.32-1.72] | 0.48 |  |  |
| Pathogen is other (vs. gram negative bacilli) | 1.09 [0.57-2.12] | 0.78 |  |  |
| Respiratory rate (per 1/min increase)* | 1.01 [0.96-1.04] | 0.97 |  |  |
| Use of accessory respiratory muscles * | 5.63 [2.83-11.82] | <0.001 | 12.8 | 3 |
| pH (for each 0.1 decrease) * | 1.51 [1.09-2.97] | 0.02 | 3.8 | 7 |
| PaCO2 (for each 1 mmHg increase)* | 1.02 [0.99-1.04] | 0.26 |  |  |
| Pa02/Fi02 ratio (for each 10 decrease)* | 1.02 [0.99-1.05] | 0.09 |  |  |
| Glasgow score (reference ≥14)* |  |  | 39.5 | 1 |
| 10-13 vs ≥14 | 3.03 [1.20-7.63] | 0.01 |  |  |
| <10 vs ≥14 | 39.95 [10.13-134.85] | <0.001 |  |  |
| Inability to clear tracheal secretions* | 2.64 [1.38-3.96] | 0.02 | 6.02 | 6 |
| Vasopressor (for each 1ug/kg/min increase)* | 2.34 [1.38-3.96] | 0.001 | 5.7 | 5 |
| Fluid loading (for each 1mL increase)£ | 1.00 [1.00-1.00] | 0.12 |  |  |
| Lactate (for each 1 mmol/L increase)* | 1.11 [1.01-1.21] | 0.02 | 8.6 | 4 |
|  |  |  |  |  |
| Variable effect: center | 0.50 [0.12-1.81]$ | <0.01 | 16.3 | 2 |

* Worst value recorded between H0 and H8, or between H0 and immediately before intubation if performed before H8.

£ Cumulative fluid from first hypotension to H0

$ variance estimate
